# Changes in the Tribological and Mechanical Properties of Nimonic 90 Superalloy After Irradiation with Swift Xenon Ions

**DOI:** 10.3390/ma18214876

**Published:** 2025-10-24

**Authors:** Piotr Budzyński, Mariusz Kamiński, Zbigniew Surowiec, Marek Wiertel

**Affiliations:** 1 Faculty of Mechanical Engineering, Lublin University of Technology, Nadbystrzycka 36, 20-618 Lublin, Poland; p.budzynski@pollub.pl; 2 Department of Physics, Institute of Physics, Maria Curie-Sklodowska University, Pl. Marii Curie-Skłodowskiej 1, 20-031 Lublin, Poland; zbigniew.surowiec@mail.umcs.pl (Z.S.); marek.wiertel@mail.umcs.pl (M.W.)

**Keywords:** irradiation, crystal structure, coefficient of friction, friction mechanism, wear, long-range effect, radiation annealing

## Abstract

**Highlights:**

**What are the main findings?**

- Swift Xe ion irradiation alters wear resistance of Nimonic 90.
- Long-range effect observed beyond projected ion range.
- High fluence (5 × 10^14^) induces radiation annealing and hardness recovery.

**What is the implication of the main finding?**

- Nimonic 90 is limited in tribological applications under irradiation.
- Radiation damage can both degrade and partially heal surface layers.
- Nimonic 90 remains valuable in aerospace and reactor components, but not in friction nodes.

**Abstract:**

The article presents the results of research on the effect of 160 MeV xenon ions irradiation on the mechanical and tribological properties of the Nimonic 90 superalloy. The alloy samples were irradiated with xenon ion fluences ranging from 1 × 10^14^ to 5 × 10^14^ Xe^24+^/cm^2^ at a temperature of 60 °C. The investigations revealed significant changes in the crystal structure of the material, including the formation of new phases and partial amorphisation of the surface layer, particularly pronounced at the highest irradiation fluence. Measurements of microhardness, coefficient of friction, and wear revealed a deterioration in the mechanical and tribological properties of the samples irradiated with fluences of 1.0 and 2.5 × 10^14^ Xe^24+^ ions/cm^2^, attributed to the formation of radiation-induced defects. Increased friction and wear were observed at depths greater than the predicted range of xenon ions, indicating the occurrence of a long-range effect. After irradiation with a 5.0 × 10^14^ Xe^24+^ ions/cm^2^ fluence, a radiation annealing effect was observed, leading to a partial reduction in the earlier damage and resulting in improved microhardness and reduced wear. To our knowledge, this is the first observation of a radiation annealing effect under these specific irradiation and test conditions. The findings suggest limitations in the application of the Nimonic 90 superalloy in environments exposed to intense ionizing radiation, such as nuclear reactors.

## 1. Introduction

Nickel-based superalloys are extensively used in aviation, space, nuclear, chemical, and petrochemical industries. Superalloys provide an excellent balance of strength and corrosion resistance at both cryogenic and high temperatures [1]. Nimonic 90 superalloy is known for its creep resistance and high ductility. In its composition, 20% of nickel has been replaced with cobalt, which has resulted in an increase in the solubility temperature of the γ′ phase in the alloy. Nimonic 90 superalloy has very good resistance to high temperatures so is commonly used as a material for blades and discs in gas turbines, as well as for critical components in jet engines and rocket engines [2]. Ni-based superalloys are widely used in the nuclear industry due to their resistance to radiation damage under a wide range of irradiation conditions [3]. Nimonic 90 is used for turbine blades and core guides in nuclear reactors. It exhibits high resistance to stress-corrosion cracking and creep, while retaining high strength at elevated temperatures [4,5]. It is mainly used in forged form [6]. However, its limited abrasive wear resistance restricts its applications. At the same time, irradiation has been shown to induce radiation hardening in Ni-based superalloys through the generation of dislocation loops, stabilization of γ′ precipitates, and irradiation-enhanced diffusion pathways. Recent studies demonstrated that γ′ precipitates in Haynes 282 remain remarkably stable even under heavy-ion irradiation up to 10 dpa, leading to only moderate increases in hardness and confirming their role as efficient defect sinks [7]. Other investigations on Inconel 718 revealed that Xe-ion irradiation at 160 MeV can significantly increase microhardness (by up to 87%), alter the γ/γ′/γ″ phase balance, and improve wear resistance, while also indicating the presence of a long-range effect [8]. These findings suggest that irradiation may, under suitable conditions, improve the tribological performance of Ni-based alloys. Depending on alloy chemistry and irradiation conditions, such effects may be beneficial (hardening, improved wear) or detrimental (precipitate dissolution, embrittlement), which underlines the need for detailed studies on Nimonic 90. In this context, it is of particular interest to investigate how Xe-ion irradiation affects the microstructure, microhardness, and wear behaviour of Nimonic 90.

To enhance wear resistance, costly surface treatments such as plasma nitriding [9], boronizing [10,11], and ion implantation [12,13,14,15] are applied. Irradiated ions lose energy through nuclear (S_n_) and electronic (S_e_) interactions, producing radiation defects that modify the material’s properties. These collisions produce radiation defects. Both radiation defects and irradiated ions alter the material’s properties. Typically, changes occur in the layer where the ions are stopped. When irradiating with ions of several hundred keV, the extent of changes in the target may exceed the ions’ range, as radiation defects can be displaced beyond the projected range (Rp) of the ions. Several dozen cases of this have been observed [16,17,18].

Changes in the mechanical properties of Nimonic 90 alloy after exposure to high-energy ions are not sufficiently described in the literature. The study [15] investigated the effect of 50 keV helium ion irradiation on the microhardness of aged Nimonic 90 alloy, which contains a γ′-precipitated phase. Irradiation with fluences ranging from 3 × 10^16^ to 3 × 10^18^ He/cm^2^ causes oscillatory variations in microhardness with increasing irradiation fluence.

Given the use of Nimonic 90 superalloy in nuclear devices, this study investigates the effects of Xe-ion irradiation with energies comparable to fission fragments. The irradiation energy was chosen with reference to the kinetic energy of ^235^U fission fragments, which is about 165 MeV [19,20]. Since this energy is distributed between two fragments depending on their masses, Xe ions with an energy of 160 MeV provide a suitable approximation of the damage produced by fission products in nuclear materials. Moreover, several previous studies have investigated metals irradiated with Xe ions of comparable energies (≈167 MeV), focusing on defect production and radiation-induced property changes [21,22,23,24]. The paper presents the results of research on the effect of swift xenon ion irradiation on the phase structure, microhardness and tribological properties of the Nimonic 90 superalloy.

## 2. Materials and Methods

The Nimonic 90 superalloy was supplied by Goodfellow Cambridge Ltd. (Huntingdon, UK) in the annealed condition (solution annealed at ~1100–1150 °C) with the nominal composition (wt.%): Ni (53), Cr (20), Co (18), Ti (2.5), Al (1.5), Fe (1.5). Circular samples with a diameter of 25 mm were cut from the sheets by water-jet cutting. The samples were mechanically polished using an automatic grinder-polisher with successive grades of SiC abrasive papers, followed by polishing with polycrystalline diamond suspensions of decreasing particle size. The final polishing step was performed with a 0.03 μm SiO_2_ suspension, yielding a mirror-like surface with an average roughness of Ra < 0.02 μm.

Irradiations were carried out with 160 MeV Xe^24+^ ions using an IC-100 cyclotron. To ensure a uniform ion distribution, the beam was scanned across the sample surface in both horizontal and vertical directions. The ion flux was limited to (4–8) × 10^12^ ions m^−2^ s^−1^ in order to minimize beam-induced heating. The samples were mounted in an aluminium holder extending outside the collector chamber, which was additionally cooled with an air stream. Under these conditions, the sample temperature did not exceed 60 °C during irradiation. The base pressure in the irradiation chamber was 1.1 × 10^−9^ Pa. The applied fluences were 1 × 10^14^, 2.5 × 10^14^, and 5 × 10^14^ ions cm^−2^, each delivered in a single step.

Micrographs of the wear track and the surface of the counterbody were captured using a scanning electron microscope (SEM) utilized for this study was the TESCAN Vega 3LMU (Tescan Group, a.s., Brno–Kohoutovice, Czech Republic) equipped with the AZtecEnergy advanced EDX microanalyzer from Oxford Instruments (High Wycombe, UK). The chemical composition of the alloy was determined using an X-ray microanalyser. Phase identification was performed by grazing incidence X-ray diffraction (GXRD) with an Empyrean diffractometer (Malvern Panalytical Ltd., Malvern, UK) equipped with a PIXcel 1D silicon strip detector (255 channels, 3.5° 2θ coverage). The use of this configuration enabled rapid data collection with high statistical quality. After monochromation, the Cu Kα radiation wavelength was set to 0.1541 nm.

The coefficient of friction and the degree of wear were measured during tribological tests conducted under technically dry friction conditions with the Anton Paar NTR^2^ tribometer (CFM Instrument) (Anton Paar GmbH, Graz, Austria). The tests were conducted in compliance with the ASTM G99-17 Standard Test Method for Wear Testing with a Pin-on-Disk Apparatus [25]. The temperature during the test was 22 °C and the relative humidity was 40%. The counterbody was a tungsten carbide (WC-6Co) ball with a diameter of 1 mm. The load applied in the tests was 0.4 N. Each sample was tested under the same tribological test parameters: the sample rotated at a frequency of 1 Hz, the friction track had a diameter of 20 mm, and the linear friction speed was 69 mm/s. In each case, the test length was 15,000 rotations, which corresponded to a friction distance of 942 m. Wear track measurements were performed using a Taylor-Hobson (Taylor Hobson Ltd., Leicester, UK) mechanical profilometer. Twenty cross-sectional profilometric measurements of the wear track on the sample surface were performed around the entire circumference of the wear track.

Microhardness was determined based on fifteen indentations for each load of 1 gf, 5 gf, and 10 gf on each test sample. The tests were performed using the Vickers method in accordance with the ASTM E384-22 standard [26], using a Future-Tech FM-800 (Kawasaki, Japan) microhardness tester. For Vickers hardness test, the indenter has a pyramidal with a square base shape with apex angle equal to 136°. In all tests the loading time was fixed at 10 s.

## 3. Results

**Figure 1:**
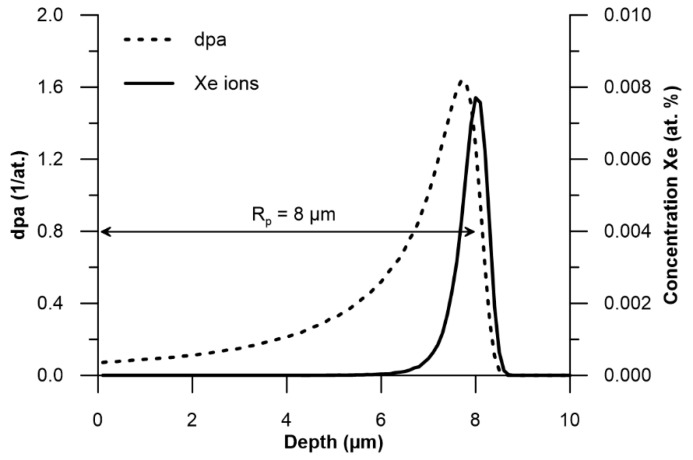
Simulation of the effects of irradiation of Nimonic 90 superalloy with swift xenon ions at a fluence of 5 × 10^14^ Xe^24+^ ions/cm^2^. The depth distribution of ions and the estimated number of displacements per atom (dpa) are shown.

Figure 1 shows the calculated distribution of xenon ions and vacancies created during collisions with target atoms. It is assumed that the distribution of radiation defects along the depth of the sample reflects the distribution of vacancies. The calculations used the SRIM programme [27]. Calculation of the range of xenon ions with an energy of 160 MeV in the Nimonic 90 superalloy was carried out using effective threshold displacement energies [28]. The projected range of xenon ions (Rp) is 8 µm. During elastic collisions with xenon ions, the positions of the atoms within the crystal lattice change. The interaction of ions with the target can be described in terms of dpa (displacement per atom), providing information about the fluence and energy of the ions, as well as the binding energy of target atoms. The dpa value indicates the potential number of atomic displacements that can occur during target irradiation leading to defect cluster formation and microstructural evolution [29]. Figure 1 illustrates the dpa values at various depths within the target.

### 3.1. Crystalline Structure

**Figure 2:**
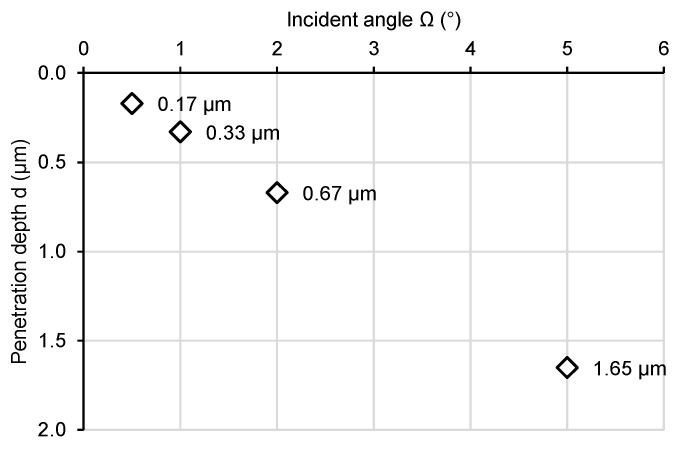
The depth of penetration of X-rays.

The crystal structure of Nimonic 90 superalloy was analysed using the GXRD method. By varying the angle of incidence (Ω) of X-rays on the sample, we alter the thickness of the measured layer. The effective penetration depth for each angle was calculated according to [30]. The angles of incidence obtained, along with the corresponding calculated X-ray penetration depths, are shown in Figure 2.

**Figure 3:**
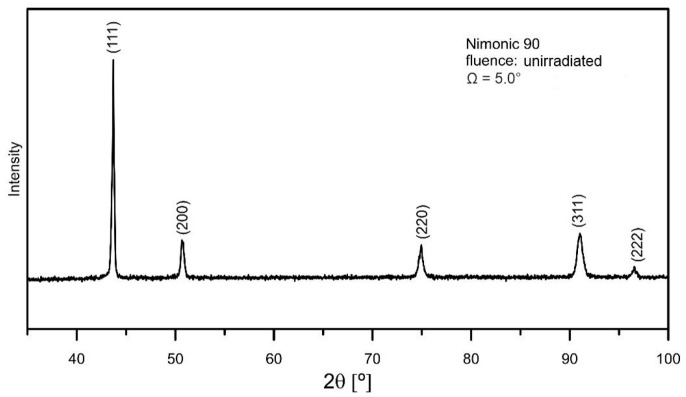
GXRD spectrum of unirradiated Nimonic 90 superalloy.

The diffractogram of unirradiated Nimonic 90 superalloy features only peaks characteristic of the γ austenite—Figure 3. It displays five lines typical of the γ phase: (111)—43.7°, (200)—50.9°, (220)—75.0°, (311)—91.3° and (222)—96.7°.

**Figure 4:**
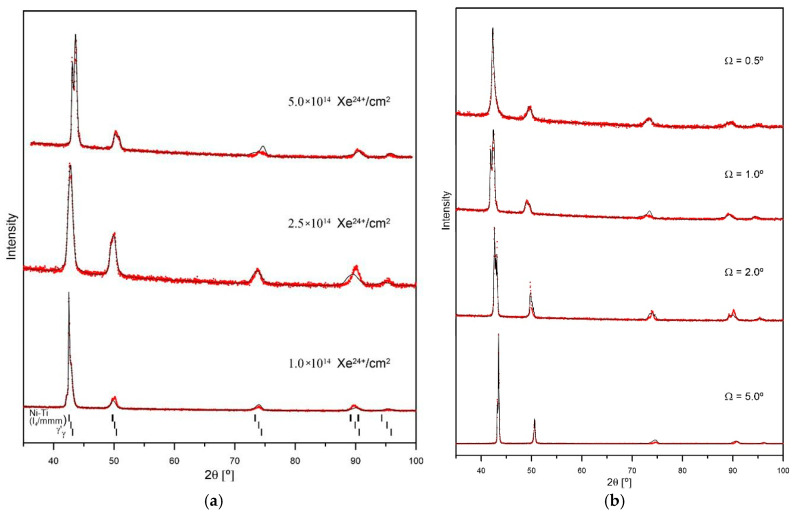
GXRD spectra of Nimonic 90 after Xe^24+^ irradiation: (**a**) 0.33 µm surface layer (Ω = 1.0°) at fluences of 1.0, 2.5 and 5.0 × 10^14^ ions/cm^2^; (**b**) different incidence angles Ω at a fluence of 5.0 × 10^14^ ions/cm^2^.

After irradiation, new phases: γ′ and β appear—Figure 4a. A higher irradiation fluence results in greater changes to the crystal structure. The contribution of the new phases to the overall spectrum increases. Irradiation at fluences of 2.5 and 5.0 × 10^14^ ions/cm^2^ promotes the amorphisation of the surface layer, which is 0.33 μm thick.

**Figure 5:**
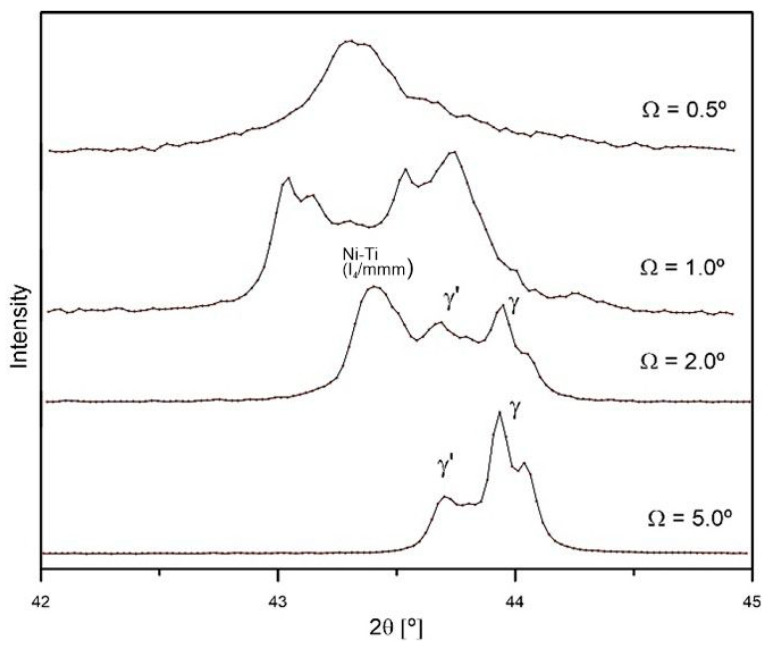
A section of the GXRD spectrum of Nimonic 90 superalloy after irradiation with a fluence of 5.0 × 10^14^ ions/cm^2^.

Irradiation with a fluence of 5.0 × 10^14^ ions/cm^2^ causes greater changes in the crystal structure of the surface layers of the samples (Figure 4b). Figure 5 presents an enlarged section of the GXRD spectrum near an angle of 43.5°. The peaks of the γ, γ′, and β phases are visible. The width of the peaks increases as they approach the sample surface. The broadening of the peaks correlates with the energy deposited during irradiation.

### 3.2. Tribological Measurements

#### 3.2.1. Friction

**Figure 6:**
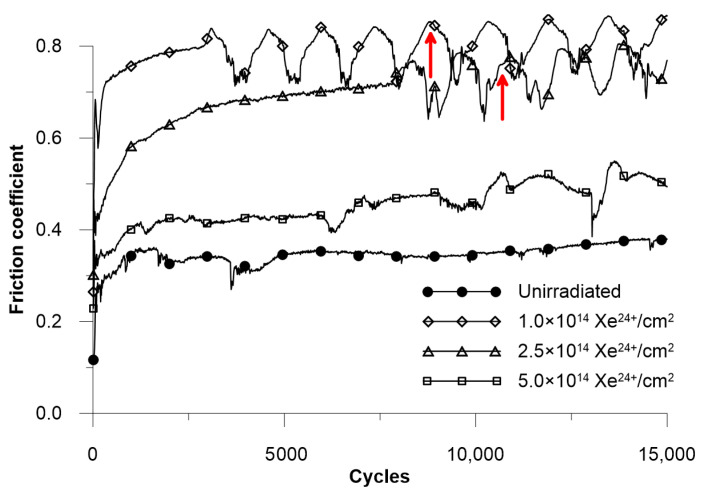
The coefficient of friction for Nimonic 90 superalloy. Arrows (↑) mark the number of cycles at which the worn track depth matches the xenon range ions.

During tribological testing, friction coefficient values were recorded, and the results are presented in Figure 6. The coefficient of friction for the unirradiated sample remains around 0.35 after 1000 measurement cycles and fluctuates only slightly during the test. Irradiation increases the coefficient of friction. A fluence of 1.0 × 10^14^ ions/cm^2^ causes a twofold rise in this value. After 2000 measurement cycles, consistent fluctuations in the coefficient of friction are observed, with an amplitude of about 0.1. These fluctuations result from the accumulation of worn material in front of the moving counterbody, which is periodically shed from the friction track once enough has built up. For the sample exposed to the highest fluence, this effect is less noticeable which results in lower sample wear and thus lower quantities of wear debris. As the irradiation fluence increases, the coefficient of friction rises less compared to unirradiated samples, and the fluctuations decrease. Irradiation with the maximum fluence (5.0 × 10^14^ ions/cm^2^) produces the smallest increase in the coefficient of friction (relative to the unirradiated sample), rising by 0.1 to reach a final value of 0.45.

**Figure 7:**
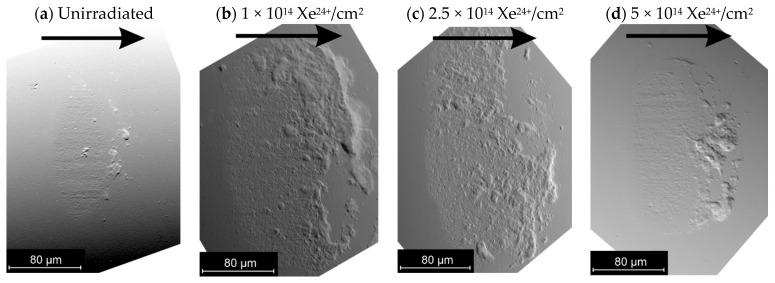
SEM images of the WC (6%Co) counterbody surface after 15,000 cycles of tribological testing. The arrows (→) indicate the direction of the counterbody’s movement relative to the sample. Magnification 1000×.

Figure 7 shows SEM images of the counterbody surfaces after the tribological test. During testing, wear particles were deposited on the counterbody surface and stayed within the friction zone. Due to friction and the generation of heat, they became plasticised but did not melt. The estimated temperature rise in Nimonic 90 wear particles during the tests was significantly lower than the melting point of the superalloy (1310–1370 °C). Fluctuations in the friction coefficient are caused by the movement of accumulated wear particles in front of the sample and their periodic removal from the wear track.

The tribological tests revealed that abrasive wear of the unirradiated sample was dominant, as shown by scratches on the counterbody—Figure 7a. After irradiating the sample with fluences of 1 and 2.5 × 10^14^ Xe^24+^/cm^2^, frictional products adhering to the worn surface of the counter sample due to adhesive interaction could be seen. Parallel wear tracks on the counterbody after irradiation with a fluence of 5 × 10^14^ Xe^24+^/cm^2^ suggest that abrasive wear remained the primary mechanism, likely due to an increase in the sample’s microhardness.

#### 3.2.2. Tribological Wear

**Figure 8:**
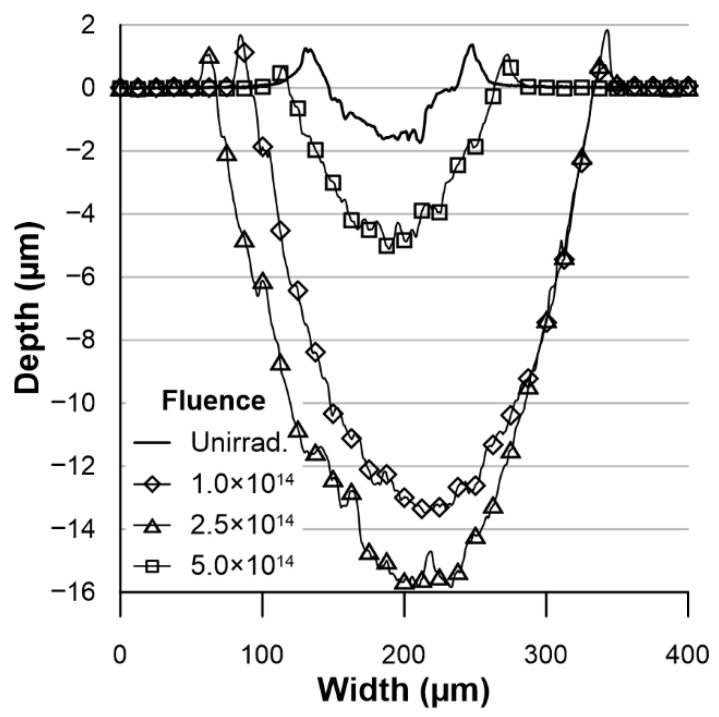
Wear track profiles on sample surfaces: both non-irradiated and irradiated.

**Figure 9:**
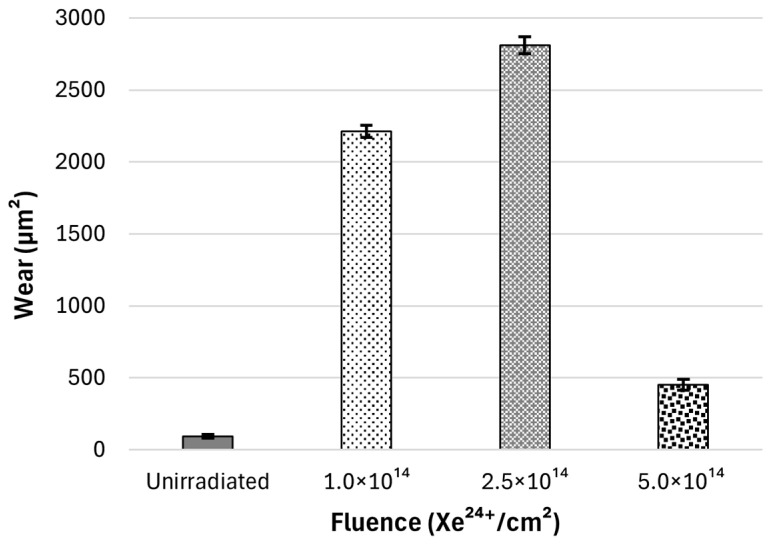
Sample wear after 15,000 cycles of tribological testing on a pin/ball-on-disk tribotester.

The wear of the tested samples after tribological tests was calculated based on the profilograms obtained (Figure 8). The cross-section of the wear track for all samples has a shape consistent with the geometry of a counterball with a diameter of Ø1.0 mm. On this basis, it can be concluded that the average wear values of the samples shown in Figure 9 represent the total wear of the friction pair. Irradiation increases the wear of the samples to varying degrees. The greatest increase in wear of irradiated samples compared to unirradiated samples occurs at an irradiation fluence of 2.5 × 10^14^ Xe^24+^/cm^2^. The smallest increase in wear occurs at a fluence of 5 × 10^14^ Xe^24+^/cm^2^.

### 3.3. Microhardness

**Figure 10:**
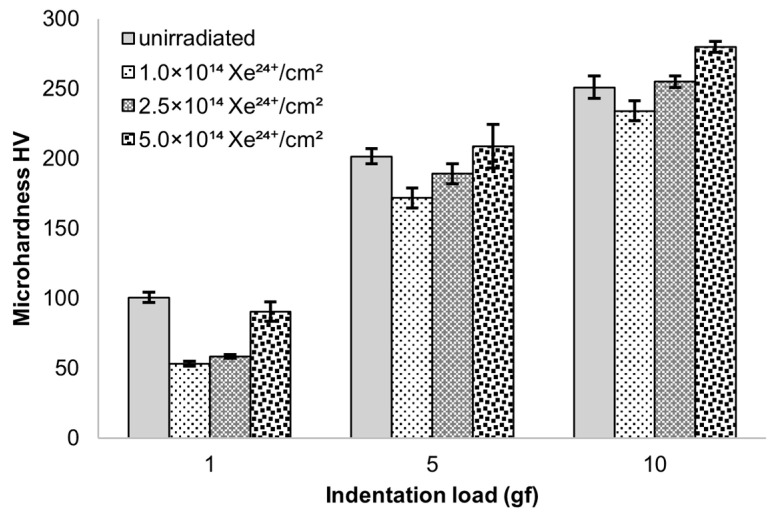
Microhardness of samples made from Nimonic 90 alloy. The diagram shows the standard error of the mean microhardness value.

Microhardness measurements were conducted on Nimonic 90 alloy (Figure 10). Irradiation decreases the microhardness of the surface layer, approximately 1 μm thick, as measured at a load of 1 gf. This is clearly evident for samples irradiated with fluences of 1.0 and 2.5 and 5.0 × 10^14^ Xe^24+^/cm^2^. The reduction in microhardness compared to unirradiated samples is 47%, 42%, and 10%, respectively.

At higher loads of 5 and 10 gf, the differences in microhardness between irradiated and reference samples were relatively small (<15%), yet systematic trends can be distinguished. At 5 gf, the lowest fluence (1 × 10^14^ ions/cm^2^) reduced hardness, the intermediate fluence (2.5 × 10^14^ ions/cm^2^) produced values close to the unirradiated alloy, while the highest fluence (5.0 × 10^14^ ions/cm^2^) slightly increased hardness. A similar tendency is observed at 10 gf, where irradiation with 5.0 × 10^14^ ions/cm^2^ resulted in a noticeable hardening compared to both the reference and the other irradiated conditions.

### 3.4. Chemical Composition

**Figure 11:**
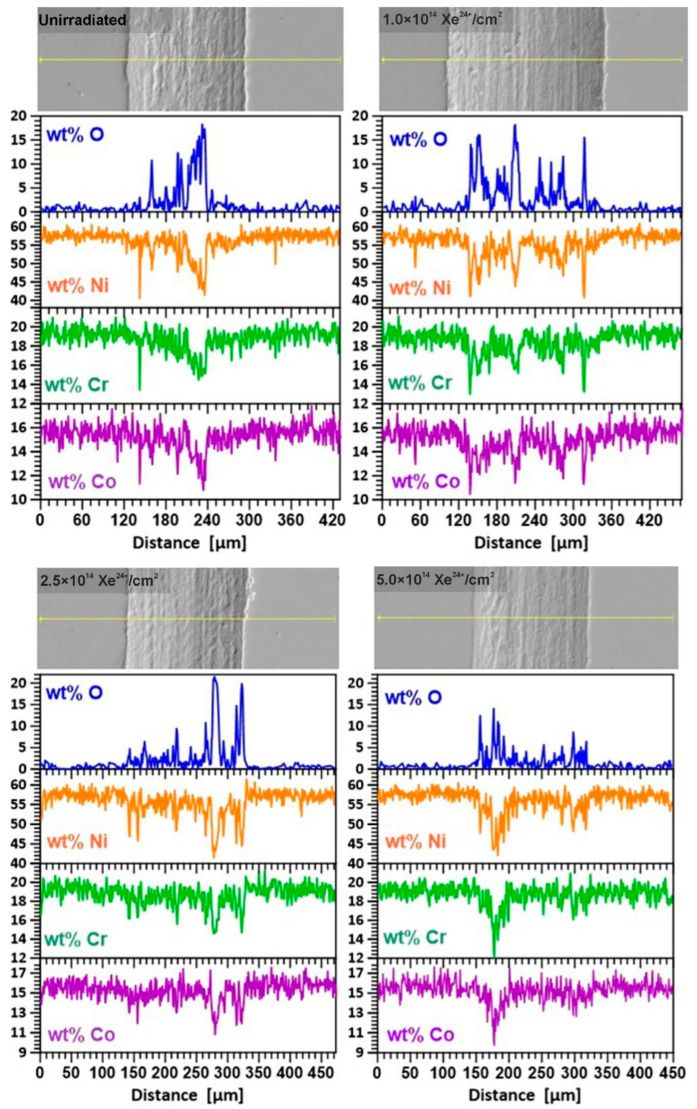
SEM image of wear tracks on samples made of Nimonic 90 superalloy after tribological testing lasting 15,000 cycles and EDX measurement results for oxygen, Ni, Cr, and Co content.

Figure 11 displays micrographs of the wear. It should be noted that tribological tests were conducted in a normal atmosphere, and the oxygen contained therein promoted the formation of oxidation effects. A line has been marked along which the content of oxygen and the main components was measured: nickel, chromium and cobalt.

The wear of samples during tribological tests is a complex phenomenon. In all cases, a similar proportion of oxidative wear appears alongside other types of wear. The core alloy components—nickel, chromium, and cobalt—are worn in comparable proportions at different points along the designated section at the bottom of the track. The lack of increased wear in any alloy component indicates there is no displacement (diffusion) of elements within the friction zone. We observe the transfer of sample material to the counterbody—Figure 7b,c.

Before irradiation, abrasive and adhesive wear are the primary factors affecting wear. After irradiation, scuffing becomes more prominent. Scuffing is particularly noticeable in the sample irradiated with the smallest fluence. This observation aligns with the findings of [31], which investigated Inconel 750. The process is facilitated by a local temperature increase during friction. Inside the worn marks on the irradiated samples, a glaze layer made of Ni and Cr oxides (NiO, Cr_2_O_3_) and a mixture of Ni(Fe, Cr)_2_O_4_ oxides can be seen. The friction products extracted from the trace were further investigated by Raman spectroscopy. The results are consistent with the preliminary EDS results.

## 4. Discussion

Irradiation of Nimonic 90 superalloy results in significant changes to its crystal structure, as well as its tribological and mechanical properties. The most significant changes in the crystal structure occur when a thin surface layer, approximately 0.3 µm thick, is irradiated (Figure 2 and Figure 4). This is evidenced by the appearance of the GXRD spectrum measured for an X-ray incidence angle of 1°—Figure 4a. In thicker layers, changes in crystal structure are smaller (Figure 4b). As the thickness of the tested layer increases, the changes in the crystal structure decrease. At a depth of about 0.7 µm (Ω = 2°), the number of new phases formed as a result of irradiation decreases significantly The appearance of γ, γ′, and β phases after irradiation, especially at higher ion doses, is a characteristic phenomenon for Ni-based superalloys [32,33]. It is this phenomenon that is considered one of the basic features that make these alloys widely used in the nuclear industry which provide dispersion enhancement and stabilise the microstructure under radiation. These ordered phase separations act as traps for radiation defects, preventing their aggregation into larger defect structures [34].

**Figure 12:**
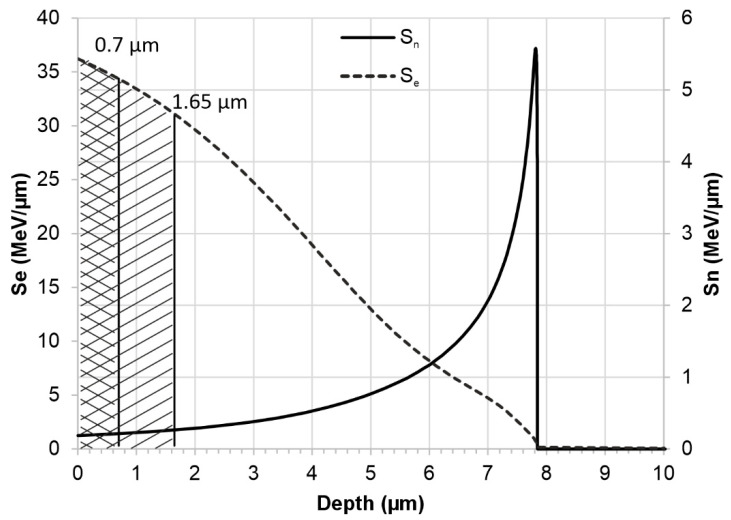
Stopping power of swift xenon ions in the Nimonic 90 target. The simulations were performed using the SRIM-2013 software package [27].

Partial amorphisation of the crystal structure after irradiation occurs in a layer approximately 0.7 μm thick. It takes place during the electron stopping phase, when the incident ion interacts with the electron system of the target and deposits energy ΔS_e_ of approximately 35 MeV/μm in this layer—Figure 12. The observed amorphization of the surface layer structure at high irradiation doses, in this case 2.5 and 5.0 × 10^14^ Xe^24+^/cm^2^, is a very common effect of the impact of heavy ions with high irradiation energy [12,35,36]. The change in the crystal structure is a collective process involving at least one lattice cell and requires the deposition of significant energy. At the end of the ion range, nuclear stopping (S_n_) occurs as a result of collisions with atomic nuclei. A small amount of energy ΔS_n_ ≤ 5.7 MeV/μm is deposited into the sample, which does not affect the friction coefficient—Figure 6.

As mentioned in the introduction, the nickel-based superalloy Nimonic 90 is characterised by reduced tribological wear resistance. However, studies show that tribological wear resistance changes under the influence of high temperatures, which cause the formation of a protective oxide layer, which is an advantage for this alloy, which is intended for use at high temperatures [37]. Tribological tests conducted on Nimonic 90 superalloy samples after irradiation indicate that their resistance to frictional wear decreases as a result of heavy ion exposures. A notable rise in wear and friction coefficient is observed in irradiated samples compared to unirradiated ones. After irradiation with a fluence of 1 × 10^14^ Xe^24+^/cm^2^, and 2.5 × 10^14^ Xe^24+^/cm^2^ a large increase in the coefficient of friction is observed—Figure 6—and a significant increase in wear—Figure 9. This indicates a completely different reaction of the alloy compared to the results obtained for the Inconel 718 superalloy, where a significant reduction in the coefficient of friction and tribological wear was observed after the same irradiation [8].

Analysis of the wear track depth and the friction coefficient curve (Figure 6) indicates that the tribological process was created mainly by the susceptibility of the sample to wear, as the tungsten carbide counter ball did not undergo measurable wear. The cross-sectional shape of the wear mark corresponded to the countersample geometry. Notably, even after the wear track depth exceeded the projected range of xenon ions of ~8 µm (Figure 1), the friction coefficient did not show any significant change compared to the period during which the layer directly exposed to Xe^24+^ ions was worn away. For the sample irradiated with a fluence of 2.5 × 10^14^ ions/cm^2^, the depth of the wear track reached almost 16 µm—nearly twice the ion range—while the friction coefficient stabilized after the running-in period and oscillated around a constant value, with variations caused only by the presence of wear debris. This behaviour confirms that the irradiation effects extend beyond the projected ion range, a phenomenon previously reported after irradiation with ions of several hundred keV energy [16,17,38,39] and commonly referred to as the long-range effect. In the present study, this effect is observed after irradiation with 160 MeV xenon ions at fluences of 1.0 and 2.5 × 10^14^ ions/cm^2^. However, it was not evident for the fluence of 5.0 × 10^14^ ions/cm^2^, as the wear was significantly reduced and the wear track depth remained smaller than the xenon ion range during the measurement cycles. Usually, the long-range effect has positive aspects—reduced wear and increased microhardness. However, in the case of Nimonic 90 superalloy, irradiation with fluences of 1.0 and 2.5 × 10^14^ Xe^24+^/cm^2^ deteriorates the tribological and mechanical properties, increasing the coefficient of friction and decreasing microhardness and resistance to friction wear, even at depths beyond the irradiated xenon range.

The discussed changes in tribological resistance correlate with the observed changes in microhardness of the tested samples (Figure 10). The most significant differences in microhardness are noticeable for the lowest load value of 1 gf. In this case, the average indentation depth was 0.7 µm. This value is approximately 10 times smaller than the thickness of the implanted layer, which makes it possible to eliminate the influence of the core hardness of the material on the microhardness measurement of the surface. The results indicate a significant reduction in microhardness for fluences of 1.0 and 2.5 × 10^14^ Xe^24+^/cm^2^ which, as can be seen from the wear results, directly affected the wear resistance of the material. A significant decrease in average microhardness was observed at a fluence of 1.0 × 10^14^ Xe^24+^/cm^2^ for all indentation loads, indicating the strongest defect accumulation in the surface layer as a result of xenon ion irradiation. With increasing fluence, partial recombination of radiation-induced defects occurs due to the additional energy deposited by implanted ions. Irradiation with a fluence of 2.5 × 10^14^ Xe^24+^/cm^2^ causes a slightly smaller increase in the coefficient of friction compared to the unirradiated sample than in the case of the sample irradiated with the lowest fluence, but in turn results in greater wear. The alterations in the crystal structure and the decrease in microhardness are also less significant compared to the unirradiated material sample. These changes occur in a layer approximately 1.65 μm thick, where slightly less energy is deposited during the electron stopping phase during irradiation, resulting in approximately ΔS_e_ = 33 MeV/μm. As a consequence, irradiation with a fluence of 5.0 × 10^14^ Xe^24+^/cm^2^ leads to an increase in both microhardness and tribological wear resistance. This effect can be attributed to defect recombination and dynamic annealing processes in the surface layer, promoted by the high energy density and transient temperature rise induced by the ion beam [40,41,42]. Such non-monotonic changes in hardness as a function of fluence have also been reported for Nimonic 90 under He-ion irradiation [15], where oscillatory variations in microhardness were observed. This confirms that competing processes of defect accumulation and radiation-induced recovery may govern the mechanical response of irradiated Ni-based alloys.

Irradiation with the highest fluence (5 × 10^14^ Xe^24+^/cm^2^) slightly increases the coefficient of friction and wear, and enhances microhardness. The notable enhancement in tribological properties and the increase in microhardness of samples irradiated at the highest fluence, compared to those exposed to lower fluences (1.0 and 2.5 × 10^14^ Xe^24+^/cm^2^), is attributed to the annealing of radiation defects formed during irradiation at lower fluences. The effect of radiation annealing has been observed many times [43,44].

## 5. Conclusions

1. Tribological test results indicate that Nimonic 90 superalloy should not be used as a structural material in friction nodes exposed to environments with high fluence of swift ions.
2. Measurements of the coefficient of friction and wear show that irradiation with swift xenon ions changes the properties of Nimonic 90 superalloy at a depth more than twice the depth of ion penetration. Changes in the target’s properties at depths beyond the ion penetration are referred to as long-range effects.
3. To our knowledge, under the present irradiation and test conditions, irradiation with lower fluences of fast ions (1.0 and 2.5 × 10^14^ Xe^24+^/cm^2^) led to an increase in the coefficient of friction and wear, whereas at the highest fluence (5.0 × 10^14^ Xe^24+^/cm^2^) a partial reversal of these effects was observed.
4. The smallest deterioration in tribological properties after irradiation with swift xenon ions is seen in samples irradiated with a fluence of 5 × 10^14^ Xe^24+^/cm^2^. In this case, there is even an increase in the microhardness of the samples, and the resistance to friction wear and the coefficient of friction remain similar (slightly higher) to those of unirradiated samples. However, this fact has no practical application for neutron-irradiated devices operating under real conditions. Before the radiation-induced healing of defects occurs due to irradiation and the tribological properties improve as a result of increased total irradiation fluence, the device will operate for a long time under adverse conditions, leading to deterioration of its tribological properties.

## Data Availability

The data presented in this study are available on request from the corresponding author.
